# A Preconscious Neural Mechanism of Hypnotically Altered Colors: A Double Case Study

**DOI:** 10.1371/journal.pone.0070900

**Published:** 2013-08-05

**Authors:** Mika Koivisto, Svetlana Kirjanen, Antti Revonsuo, Sakari Kallio

**Affiliations:** 1 Department of Psychology, University of Turku, Turku, Finland; 2 Centre for Cognitive Neuroscience, University of Turku, Turku, Finland; 3 Department of Behavioural Sciences, University of Helsinki, Helsinki, Finland; 4 School of Humanities and Informatics, University of Skövde, Skövde, Sweden; Monash University, Australia

## Abstract

Hypnotic suggestions may change the perceived color of objects. Given that chromatic stimulus information is processed rapidly and automatically by the visual system, how can hypnotic suggestions affect perceived colors in a seemingly immediate fashion? We studied the mechanisms of such color alterations by measuring electroencephalography in two highly suggestible participants as they perceived briefly presented visual shapes under posthypnotic color alternation suggestions such as “all the squares are blue”. One participant consistently reported seeing the suggested colors. Her reports correlated with enhanced evoked upper beta-band activity (22 Hz) 70–120 ms after stimulus in response to the shapes mentioned in the suggestion. This effect was not observed in a control condition where the participants merely tried to simulate the effects of the suggestion on behavior. The second participant neither reported color alterations nor showed the evoked beta activity, although her subjective experience and event-related potentials were changed by the suggestions. The results indicate a preconscious mechanism that first compares early visual input with a memory representation of the suggestion and consequently triggers the color alteration process in response to the objects specified by the suggestion. Conscious color experience is not purely the result of bottom-up processing but it can be modulated, at least in some individuals, by top-down factors such as hypnotic suggestions.

## Introduction

Suggestions given with or without hypnosis may alter conscious color perception and modify neural activity in color processing areas of the brain [1]–[3]. Furthermore, suggestions to see specific objects in certain colors posthypnotically (i.e., after hypnosis has been cancelled) may selectively alter their perceived color [4]. The neural mechanisms of such alterations are not known. We hypothesized that for a posthypnotic suggestion to rapidly alter the perceived color of a subset of objects selectively, some mechanism must compare the early bottom-up signal to the suggested content in order to trigger the color alteration process before the object enters consciousness. High-frequency neural oscillations provide a mechanism for rapid comparison and communication between distant brain areas. For instance, the early evoked gamma-band response is known to reflect automatic matching of bottom-up signals with memory contents about 100 ms after the stimulus-onset [5]. Thus, object-specific posthypnotic alterations in color perception might involve an early high-frequency mechanism that compares the bottom-up input to the content of the suggestion in order to identify the objects relevant for the suggestion.

We investigated this hypothesis by measuring evoked oscillatory activity in response to different shapes presented in a rapid sequence. The color of the shapes had to be identified after a posthypnotic color alteration suggestion, which was targeted to one of the shapes in turn (e.g., “all triangles are red”). In a simulation condition, the participants were instructed to behave as if having received such a suggestion. The subjective experiences in response to suggestions vary largely even among highly hypnotizable individuals, indicating that they should not be considered a homogenous group [6], [7]. Therefore we conducted a double case study and focused on two highly hypnotizable participants, TS-H and RM, who performed the task several times.

## Materials and Methods

### Ethics Statement

The research was conducted according to the ethical standards of the American Psychological Association (APA) and approved by the Ethics Committee of the University of Turku, Finland (statement 18/2011). All subjects gave their written informed consent for participation in the study.

### Participants

TS-H is a 45-year old, right-handed, healthy woman with no psychiatric or neurological history. She reports vivid visual and acoustic hallucinations in response to suggestions both during hypnosis and posthypnotically (for a description of a posthypnotic suggestion, see [8]). In addition, she experiences spontaneous posthypnotic amnesia and is typically unaware of the suggestions given during hypnosis. TS-H scores a full 12 points in the two most widely used scales measuring hypnotic suggestibility (Harvard Group Scale Of Hypnotic Susceptibility Form A [HGSHS-A] [9] and Stanford Hypnotic Susceptibility Scale Form C [SHSS-C] [10]. She can be hypnotized and returned into a normal waking state by using a one-word induction (see [8] for a video-clip of the procedure). Her brain functioning [11]–[13] and automatic eye-movements [8] are immediately altered by hypnosis. It is unknown what proportion of highly hypnotizable participants are similar to TS-H, but they are very rare and difficult to find with the standard screening procedures [14].

RM is very highly hypnotizable 40-year old, right-handed, healthy woman without psychiatric or neurological history. She scores 12 and 9 points in HGSHS-A and SHSS-C, respectively. Although RM is highly hypnotizable, she does not experience visual hallucinations in response to hypnotic suggestions. She too can be hypnotized and returned into a normal waking state by using a one-word induction.

### Stimuli and the Task

**Figure 1:**
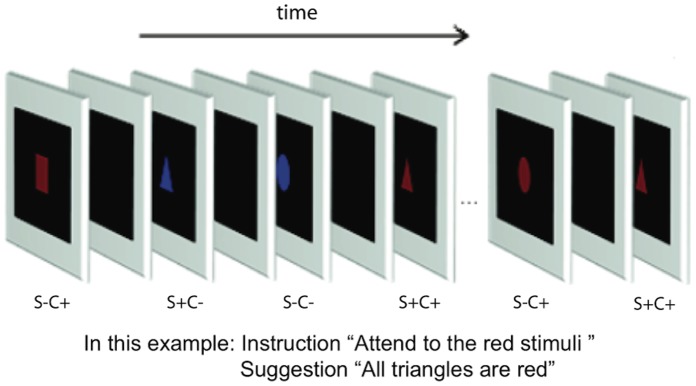
Stimulation sequence. The stimuli were presented in random order for 24 ms in red or blue with 800–1200 ms interstimulus-interval. The target color was either red or blue. In the posthypnotic conditions, a suggestion that one of the shapes is always presented in a specific color (e.g., “all triangles are red”) was given. In the simulation condition, the task was to behave as if having received such suggestion. (S+C+ = relevant shape, target color; S+C− = relevant shape, nontarget color; S−C+ = irrelevant shape, target color; S−C- = irrelevant shape, nontarget color).

The stimuli were squares, triangles and circles, presented either in red or blue color with E-prime software in random order in the centre of a CRT screen for 24 ms (85 Hz, 1024×768 pixels resolution; Fig. 1). The interstimulus-interval varied randomly between 800–1200 ms. The luminance was 12.9 cd/m^2^ for red and blue colors and 0.2 cd/m^2^ for the black background. From the viewing distance of 150 cm, the size of the stimuli was about 2.5°×2.5°.

Each participant performed a total of 48 stimulus blocks: 12 blocks in a behavioral session and 36 in three EEG sessions. Each block involved 216 trials (36 presentations of each of the six combinations of three shapes and two colors). The attended-to target color was red in 50% of the blocks and blue in the other 50%. To avoid interference from one target color to another, the target color was changed only once in each session [red (or blue) was attended to in the first six blocks and blue (or red) in the last six blocks].

We measured behavioral responses in a separate session to study the phenomenal effects of the posthypnotic suggestion on subjective (conscious) color perception. During the behavioral sessions, the participants pressed a button for every stimulus they saw in the color that was specified at the beginning of the stimulus block as the attended-to target color. In the posthypnotic conditions, the participants were given the posthypnotic suggestion that a specific shape will appear in the target color (e.g., “all triangles are blue”). In the simulation conditions, the participants were asked to behave as if they had received the suggestion and as if they actually saw the shape in the target color. We used the simulation condition instead of a condition where suggestions are given without hypnosis [2], because we know from previous testing that TS-H and RM do not report color changes without hypnosis but with otherwise identical suggestions and a stable target.

During the EEG sessions, the task was identical to that in the behavioral sessions, with the exception that the participants were asked to covertly count the targets and to respond overtly only to every 10^th^ target. This procedure was adopted in order to keep the EEG data clean from possible motor artefacts (the trials with a button press were eliminated from EEG analyses). It remains, however, possible that covert counting might also produce artefacts (selectively to target stimuli), although probably to a lesser extent than responding to all the targets. In any case, some kind of task requiring responding was needed to keep the participants attention on the stimuli. We carefully instructed the participants to avoid movements of mouth or lips during the task.

### Hypnosis and Suggestions

A one-word induction of hypnosis [8] was applied for both participants. All the hypnotic suggestions were posthypnotic, that is, under hypnosis the participants were given suggestions that they would see particular shapes in specific color (e.g., seeing squares as red) when performing the task in a full waking state.

The participants performed half of the stimulus blocks in the posthypnotic condition and half in the simulation condition. The order of the posthypnotic and simulation conditions was altered between blocks. Each shape was targeted by the suggestion (i.e., was the suggestion-relevant shape) equally frequently across the conditions.

In the posthypnotic condition, the color alteration suggestion was given in hypnosis. After this suggestion, the participants were given a suggestion for posthypnotic amnesia that they would not remember the color suggestion in the normal waking state. After this, the hypnosis was cancelled and the stimulus block was started. Thus, all the stimulus blocks were performed in the normal waking state. After each posthypnotic stimulus block, hypnosis was induced again and the posthypnotic suggestion was cancelled.

Although the presence of posthypnotic amnesia to the suggestions cannot be ultimately proved, we tried to verify it by asking the participants after hypnosis: “What did we talk about during hypnosis?”, “Did you get any instructions during hypnosis?” Both participants maintained that they did not remember what happened during hypnosis.

### Electrophysiological Recording

EEG was recorded using Ag/AgCl electrodes from the international 10/20 system sites Fp1, Fp2, F3, F4, F7, F8, Fz, P3, P4, Pz, C3, C4, Cz, T3, T4, T5, T6, O1, and O2. Nose was used as a reference location and an electrode between Fz and Cz as the ground. Horizontal and vertical eye movements were monitored with electrodes placed 1.5 cm to the right of and below the right eye, respectively. EEG was amplified by using a band pass of 0.15 to 100 Hz, with a sampling rate of 1000 Hz. The impedance was kept below 5 kΩ. Trials showing evidence of eye blinks, movements, or other artefacts in any of the electrodes (>70 µV) were rejected off-line. Also trials in which a behavioural response was given during the epoch (from −200 ms before to 800 ms after the stimulus onset) were rejected. With all these procedures, 14% of TS-H’s trials and 8% of RM’s trials were removed.

Phase-locked, evoked oscillations were analyzed with the Morlet Complex wavelet analysis of EEG, as implemented in Brain Vision Analyzer 2.0 (Brain Products, Gilching, Germany). It was performed with a Gaussian shape using a Morlet parameter *c* value of 4. This procedure was initially applied to the frequencies ranging from 15 to 75 Hz in steps of 1 Hz in order to get an overall idea about the frequency bands where interesting phenomena in processing the color or shape occur. Because all the interesting effects occurred in the beta band (see Data analyses below), we restricted the final analyses on 15–35 Hz. The wavelets were calculated on each participant’s unfiltered averaged evoked potentials for each stimulus type in epochs ranging from 200 ms before to 200 ms after the stimulus-onset. The baseline was corrected for the activity 200–0 ms preceding the visual stimulus. The wavelet analysis was performed separately for the evoked potentials in each stimulus block (18 blocks/condition/participant). In the event-related potential (ERP) analyses, a longer epoch from −200 to 400 ms was used and the waveforms were filtered with 0.05 Hz high-pass and 30 Hz low-pass.

### Data Analyses

The alpha level of 0.05 was used in statistical analyses and the reported P-values are two-tailed. The behavioral data did not pass the requirements of normal distribution (Kolmogorov-Smirnov’s test) and homogeneity (Levene’s test) for parametric tests and were analyzed with nonparametric tests. The electrophysiological data passed these requirements with minor violations and were examined with analyses of variance (ANOVAs).

The frequency of evoked activity and the time windows for their statistical analyses were selected on the bases of difference scalograms for shape and color. In order to obtain differences which were unbiased in relation to the critical experimental condition (i.e., posthypnotic vs. simulation), the results from all conditions were first pooled. The difference scalograms showed that relevant shapes (i.e., the suggestion-relevant shapes in the posthypnotic and simulation conditions pooled together) elicited the greatest difference in activity in relation to the irrelevant shapes 70–120 ms from the stimulus-onset in posterior electrodes at the central frequency of 22 Hz. Thus, the data from the occipital, parietal and posterior temporal electrodes were pooled and the statistical analyses were conducted at the central frequency of 22 Hz on the mean amplitudes in the 70–120 ms latency range.

The electrophysiological data were averaged separately for each participant’s every stimulus block (for each participant, N = 18+18) and entered into ANOVAs. The general ANOVAs involved Shape (2: suggestion-relevant vs. -irrelevant) and Color (2: target vs. nontarget) as repeated factors as well as Condition (2: posthypnotic vs simulation) and Participant (2) (in the analysis of the beta activity) as fixed factors. The effects of Shape or Color were tested separately in the different conditions when a significant interaction with Condition was observed.

## Results

### Behavioral Session

Behaviorally both participants responded to the posthypnotic suggestions although in different ways. TS-H pressed the response button in response to the stimuli presented in the suggestion-relevant shape but in nontarget color (S+C−; e.g., a blue triangle when red was the target color that required responding and “all squares are red” was the suggestion) more frequently after the color change suggestion in the posthypnotic condition (96%) than when mimicking the effects of the suggestion in the simulation condition (44%) (N = 12 stimulus blocks, Mann-Whitney *U* test, P = 0.004). Thus, after the posthypnotic suggestions TS-H reported subjective color alterations in the targeted shapes (e.g., seeing a blue triangle as red) in almost every trial but she was less able to simulate the effects of such suggestions (i.e., she pressed the response button less frequently when she had not received the suggestion but only tried to behave as if having received it). The reversed pattern was true for RM who reported less posthypnotic color alterations (31%) but performed well in the simulation (95%) (N = 12 stimulus blocks, Mann-Whitney *U* test, P = 0.004). However, further testing revealed that RM did not actually perceive altered colors after the suggestion but experienced a conflict between what she saw and what she *felt* the color was (see *Additional behavioral results of RM* below).

### Behavioral Results during the EEG Sessions

During the EEG sessions, the participants did not respond with a button press to every target (i.e., to the stimuli with the attended-to color) but counted them silently them and responded with overt button press to every 10^th^ target (to avoid motor artefacts in EEG data). Therefore their classification performance during the EEG recording can be roughly estimated by multiplying the number of their responses by ten. In the posthypnotic condition, TS-H responded to 8% of the shapes targeted by the suggestion (S+C−); the corresponding value was 3% in the simulation condition. Multiplying these values by ten suggests that in posthypnotic condition she classified about 80% of the S+C− trials according to the suggestion; in the simulation condition she classified about 30% of the S+C− according to the suggestion. RM responded to 4% of the S+C− trials in the posthypnotic condition and to 8% of the S+C− trials in the simulation condition, giving the classification estimates of about 40% and 80% in the posthypnotic and simulation conditions, respectively. For both participants, these estimated patterns are in line with the results from the behavioral sessions in suggesting that TS-H performed well in the posthypnotic condition and less well in the simulation condition, while the reverse was true for RM.

### Additional Behavioral Results of RM

RM’s responded to the suggestion-relevant shape in nontarget color (S+C−) in 31% of the trials as if the color would have been changed after posthypnotic suggestion. She reported after the behavioral and EEG sessions that sometimes she experienced a conflict between what she saw and what she *“felt”* the color is or that *“sometimes I saw the shape as red (or blue)* (i.e., in the attended-to color) *but my brain said it had a different color”*. Therefore we hypothesized that she did not experience visually the color alterations and tested her with two additional behavioral stimulus blocks of 216 trials in the posthypnotic condition.

In both of the additional blocks, blue was the attended-to color and RM was asked to respond with a button press to each target stimulus. Both stimulus blocks were performed under the posthypnotic suggestion that “all circles are blue”. The instructions for the first stimulus block stressed that she should respond only according to the color that she actually sees and to ignore totally what she feels. In this block, RM did not report any change of color from red to blue for the suggestion-relevant stimuli with the nontarget color (0%)(i.e., red circles; S+C−), while she responded correctly to the blue stimuli in 100% of the trials and incorrectly to 3% of the irrelevant nontargets (i.e., red squares and red triangles; S−C−). In other words, she did not see the red circles as blue in any of the trials. However, in the second stimulus block, she was instructed that when a conflict between what she sees and what she feels appears, she should respond only according to what she feels and not according to what she really sees. In this condition, RM responded to 75% of the red circles (S+C−), to 97% of the blue shapes and to none of the suggestion-irrelevant nontargets (i.e., red squares and triangles, S−C−). The difference in responding to the red circles (S+C−) between the two instructions was highly significant (N = 72, Pearson Chi-Square = 42.20, p<0.001).

In conclusion, RM did not experience color alterations, but the reported difference between seeing and feeling elicited by the suggestion shows that her brain was able to discriminate between the suggestion-relevant and –irrelevant shapes in the posthypnotic condition in spite of the reported posthypnotic amnesia for the suggestion. An alternative explanation of RM’s apparent conflict between seeing and feeling would state that she did not have posthypnotic amnesia and thus knew how she was expected to respond after the color alteration suggestion but that this was in conflict with what she actually saw. This alternative explanation, however, seems less likely because it leaves it open that why did RM claim to have a posthypnotic amnesia but at the same time claimed that she did not see the color alterations. If she behaved according to the demand characteristics, why did not she play the role to the very end and state also that she saw the colors as suggested? A more coherent explanation of her behavior is that she really suffered from posthypnotic amnesia and the suggestion produced the strange feelings she had.

### Evoked Beta Activity

**Figure 2:**
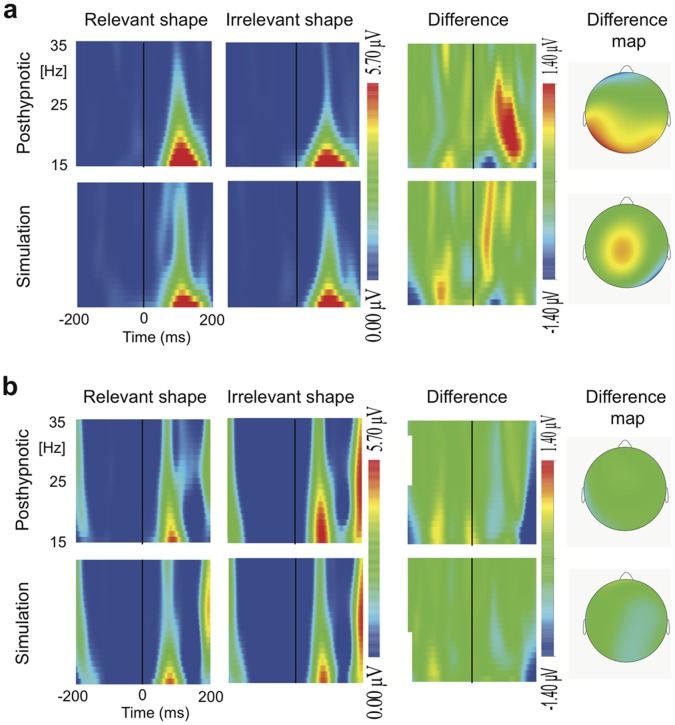
Evoked potential responses. Time-frequency representations of the evoked responses to suggestion-relevant and -irrelevant shapes and their difference scalogram in the posthypnotic and simulation conditions at 15–35 Hz over the left occipital cortex for (A) TS-H and (B) RM. The maps on the right side show the scalp distribution of the shape related difference at the central frequency of 22 Hz in the 70–120 ms post stimulus time window.

**Figure 3:**
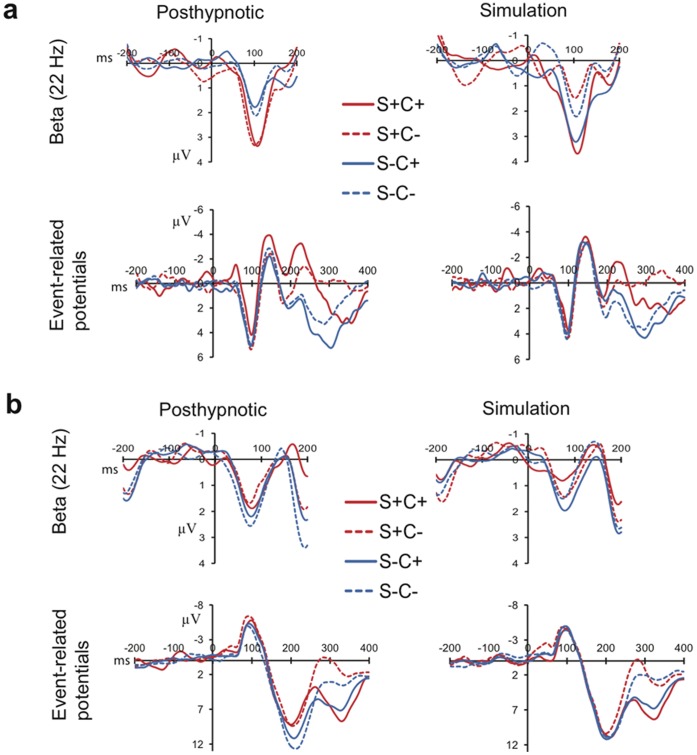
The time course of the evoked responses at 22 Hz and event-related potentials over the left occipital cortex for each stimulus type in the posthypnotic and simulation conditions for (A) TS-H and (B) RM. The difference in TS-H’s evoked beta between suggestion-relevant shapes (S+C+, S+C−) and –irrelevant shapes (S−C+, S−C−) is larger in the posthypnotic condition than in the simulation. TS-H’s event-related potentials between 200 and 300 ms show enhanced negativity/decreased positivity to the suggestion-relevant shapes relative to irrelevant shapes in the posthypnotic and simulation conditions. RM’s event-related potentials show enhanced negativity/decreased positivity to the suggestion-relevant shapes only in the posthypnotic condition 150–250 ms after stimulus-onset.

Condition (2: posthypnotic vs. simulation)×Shape (2: suggestion-relevant vs. suggestion-irrelevant)×Color (2: target vs. nontarget)×Participant (2) ANOVA was performed on the mean amplitudes of the evoked beta-band response in the 70–120 ms time-window (Fig.2 and 3; see also Fig.S1 for difference scalograms at 1–75 Hz). It revealed a Condition×Shape×Participant interaction (F_1,68_ = 4.30, P = 0.042, η_p_^2^ = .06), indicating that the participants responded differently to the suggestion-relevant shape depending on the condition. In addition, the higher order Condition×Shape×Color×Participant interaction was significant (F_1,68_ = 4.98, P = 0.029, η_p_^2^ = .07). Further analyses were performed separately on each participant’s data.

For TS-H (Fig.2a and 3a), a Condition×Shape interaction was found (F_1,34_ = 6.30, P = 0.017, η_p_^2^ = .16), showing a larger difference in the evoked beta response between the suggestion-relevant (S+C+, S+C−) and -irrelevant (S−C+,S−C−) shapes after the posthypnotic suggestion than in the simulation. In the posthypnotic condition, the relevant shapes evoked a larger response than irrelevant shapes (1.96 µV ±0.35 vs. 0.88 µV ±0.25; mean ± s.e.m.) (F_1,17_ = 6.31, P = 0.022, η_p_^2^ = .27), whereas there was no such difference during the simulation condition (0.99 µV ±0.35 vs. 1.43 µV ±0.25). The increased beta response to suggestion-relevant shapes in the posthypnotic condition was replicated across the three EEG sessions (Session×Shape: F <1, P = 0.966).

The analysis of RM’s data (Fig. 2b, and 3b) revealed a Condition×Shape×Color interaction (F_1,34_ = 5.21, P = 0.029). There was no significant effect in the posthypnotic condition. In the simulation condition the Shape×Color interaction was significant (F_1,17_ = 5.41, P = 0.033, η_p_^2^ = .24), resulting mostly from the nonsignificant (P = 0.077) attenuation of the beta response to the relevant shapes in the attended-to color (S+C+), as compared with the response to irrelevant shapes in the nontarget color (S−C−).

### Event-related Potentials

In the analyses of the event-related potentials (ERPs), we focused on selection negativity (SN) [15] in response to shape. SN is observed as enhanced negativity (or decreased positivity) to the attended-to features, as compared with the unattended features. SN begins typically 140–180 ms after the onset of the stimulus and persists thereafter for 100–200 ms [15]. Visual inspection of the grand average waveforms showed that the SN to shape began for both participants around 150 ms (notably, RM’s ERPs lack the P1 potential around 100 ms, which seems to be due to the rather early N1 that masks the P1). For TS-H the SN was the strongest about 200–300 ms after the stimulus-onset, and for RM it was clearly visible already 150–250 ms after the stimulus-onset. The ERP analyses were performed in both time windows for each participant.

The Shape (2)×Color (2)×Condition (2) ANOVA for TS-H’s data (150–250 ms) showed a significant SN, that is, enhanced negativity/decreased positivity to the suggestion-relevant shapes (0.19 µV ±0.32) relative to irrelevant shapes (1.28 µV ±0.19)(F_1,34_ = 10.40, P = 0.003, η_p_^2^ = .23); the posthypnotic and simulation conditions did not differ in SN (F <1). Similarly, in the time window (200–300 ms) where the SN was the largest for TS-H, she showed a significant SN (suggestion-relevant shapes: 0.18 µV ±0.39; irrelevant shapes: 2.23 µV ±0.21)(F1,34 = 38.05, P<0.001, η_p_^2^ = .53), without a difference between the conditions in the magnitude of SN (P = 0.292). These findings indicate that TS-H processed the suggestion-relevant shape selectively also in the simulation condition. Therefore the dissociation in the evoked beta-band response between the posthypnotic and simulation conditions cannot be explained by stating that TS-H did not follow the instructions and did not attend to the relevant shapes during the simulation condition.

The analysis of RM’s data (150–250 ms) (Fig.3b) shows a main effect for Shape (F_1,34_ = 6.88, P = 0.013, η_p_^2^ = .17). However, the Shape×Condition interaction (F_1,34_ = 5.05, P = 0.031, η_p_^2^ = .13) indicates that she showed the SN for shape, that is, increased negativity/less positivity to the suggestion-relevant shapes (3.35 µV ±0.38) relative to irrelevant shapes (4.94 µV ±0.26) in the posthypnotic condition (F_1,34_ = 9.26, P = 0.007, η_p_^2^ = .35), but not in the simulation condition (F <1). RM showed a Shape×Condition interaction (F_1,34_ = 6.59, P = 0.015, η_p_^2^ = .16) also in the 200–300 ms time window, indicating a SN for shape in the posthypnotic condition (2.64 µV ±0.37 vs. 4.70 µV ±0.30) (F_1,34_ = 17.72, P = 0.001, η_p_^2^ = .51) but not in the simulation condition (3.58 µV ±0.36 vs. 3.93 µV ±0.33) (F <1). Thus, in addition to the subjective feelings (See *Additional behavioral results of RM*), RM’s brain activity was also different depending on whether she performed under the posthypnotic suggestion or simulated the effects of the suggestion.

## Discussion

The mechanisms of hypnotic color alterations were studied by asking two very highly hypnotizable participants (TS-H and RM) to detect the colors of briefly presented shapes in the normal waking state after having been given posthypnotic suggestions in hypnosis that specific shapes will appear in altered colors. TS-H reported altered colors in the targeted shapes and her results showed that oscillatory activity in the higher beta-band correlated with the contents of the suggestion. Her evoked 22 Hz activity over the posterior cortex was enhanced in response to the suggestion-relevant shapes 70–120 ms after the stimulus-onset. When simulating the effects of suggestion, the modulation of the beta activity was not observed, although TS-H’s ERPs to the suggestion-relevant shapes showed selection negativity (SN) after 200 ms. This indicates that TS-H attended to the suggestion-relevant shapes and thus a lack of attention to the shapes during the simulation cannot explain the dissociation in the beta activity between the posthypnotic condition and the simulation.

These results converge with the view that evoked high-frequency oscillations reflect automatic matching of the input to memory representations [5], in this case to that of the posthypnotic suggestion. The matching must have occurred preconsciously because of the early latency of the effect, the immediacy of the color change, and because the participants reported having performed under posthypnotic amnesia without conscious memory of the suggestions.

A fundamental difference in our experiment in relation to previous relevant brain imaging and electrophysiological studies on color or word processing [1], [3], [16] is that here the suggestion was targeted to specific shapes in a rapid sequence of various shapes, not to all stimuli generally. Therefore the observed effects cannot be explained by a general attenuation or increase of activity in the visual cortex. Our results thus reveal tighter constraints on the architecture of the system that generates conscious visual experiences and suggest that rapid recurrent loops must be involved in conscious color perception. This fits well with recent theories of visual awareness that emphasize the role of recurrent interactions between higher and lower visual areas in binding the visual features into coherent conscious percepts [17]–[18]. Rapid detection of relevant shapes by the preconscious matching mechanism gives the suggestion a possibility to modulate the brain activity in color areas in a recurrent manner before the object is consciously perceived in its altered colors.

There are differences among highly hypnotizable individuals regarding the outcomes of the suggestions [6]. The participant RM showed altered behavioral responses and event-related potentials to suggestion-relevant shapes, but she did not report color alterations and her evoked oscillations were not influenced by the suggestions: her neural responses were not sufficient to overrun the bottom-up color signal and to produce subjective color alterations. This result is in line with previous studies suggesting major differences among highly hypnotizable individuals [19], [20]. Further studies examining participants in a case-by-case manner are needed to explain the individual variability in responses to color suggestions.

This study has also clear theoretical implications concerning the nature of hypnotically induced responding. According to some major theories of hypnosis [21], [22] suggestions given in a hypnotic context are *always* “imaginative suggestions” asking the subjects to engage in fantasies leading to subjective experiences *that they know are not objectively true*. However, it is also possible to give *deceptive* hypnotic suggestions which aim to convince the person that the world is different from the way it actually is (compare e.g. the fly hallucination suggestion in HGSHS:A [9]). In the present study we used this kind of deceptive suggestions stating that the world outside has actually changed (e.g. “…all the squares that you will see on the screen are red”). TS-H reported that she saw the suggested changes and, furthermore, she did not experience anything abnormal in her subjective experience during the task. RM on the other hand reported a curious feeling associated with the target shapes so that what she saw and what her “brain said” were mismatching. This result strongly suggests that TS-H was not aware of the physical color at all and was not merely trying to imagine the suggested color. Therefore, the concept of “imaginative suggestibility” does not seem to capture the whole range of hypnotic phenomena (see also [23]).

There are two major theoretical questions in hypnosis research, one pertaining to the nature of suggestions (are they based on voluntary imagination or not) and the other on the nature of hypnosis itself (is there a special state involved or not) [7], [22]. Our present results mostly relate to the first question, and support the view that suggestions, at least in some individuals, are different from voluntary imaginings (see also [24]), and therefore difficult to explain by the notion of imaginative suggestibility. As to the second question, our earlier studies on the same subject TS-H support the view that, at least in some individuals, a genuine hypnotic state occurs that cannot be imitated or simulated [8].

To conclude, we have shown for the first time an objective neural correlate for the influences of stimulus-specific suggestions. Although we cannot objectively verify the phenomenological reality of the subjective color experience (see e.g. [25]), the general consensus in hypnosis research is that hypnotic suggestions change subjective experiences and not merely the reports of subjective experiences [26], [27]. The effects of the suggestions must rely on brain structures and functional connections that are available in normal brain [4]. Therefore our study suggests also that normal conscious color experience is not purely the result of bottom-up processing but top-down factors can have a modulatory effect on it [28].

## Supporting Information

Figure S1 **The difference scalograms for evoked activity (suggestion-relevant minus -irrelevant shapes) at 1–75 Hz over the left occipital cortex for TS-H and RM.** The white square indicates the area where the posthypnotic suggestion influenced TS-H’s beta activity.
(EPS)
